# A multilevel examination of lifetime aggression: integrating cortical thickness, personality pathology and trauma exposure

**DOI:** 10.1093/scan/nsab042

**Published:** 2021-04-10

**Authors:** Ana E Sheehan, Nadia Bounoua, Rickie Miglin, Jeffrey M Spielberg, Naomi Sadeh

**Affiliations:** Department of Psychological and Brain Sciences, University of Delaware, Newark, DE 19176, USA; Department of Psychological and Brain Sciences, University of Delaware, Newark, DE 19176, USA; Department of Psychological and Brain Sciences, University of Delaware, Newark, DE 19176, USA; Department of Psychological and Brain Sciences, University of Delaware, Newark, DE 19176, USA; Department of Psychological and Brain Sciences, University of Delaware, Newark, DE 19176, USA

**Keywords:** aggression, cortical thickness, personality, trauma

## Abstract

Aggression represents a significant public health concern, causing serious physical and psychological harm. Although many studies have sought to characterize the etiology of aggression, research on the contributions of risk factors that span multiple levels of analysis for explaining aggressive behavior is lacking. To address this gap, we investigated the direct and unique contributions of cortical thickness (level 1), pathological personality traits (level 2) and trauma exposure (level 3) for explaining lifetime physical aggression in a high-risk sample of community adults (*N* = 129, 47.3% men). First, the frequency of lifetime aggression was inversely associated with cortical thickness in regions of prefrontal and temporal cortices that have been implicated in executive functioning, inhibitory mechanisms and socio-emotional processing. Further, aggression was positively associated with pathological personality traits (antagonism and disinhibition) and exposure to assaultive trauma. Notably, all three levels of analysis (cortical thickness, pathological personality traits and assaultive trauma exposure) explained non-overlapping variance in aggressive behavior when examined simultaneously in integrative models. Together, the findings provide a multilevel assessment of the biopsychosocial factors associated with the frequency of aggression. They also indicate that cortical thickness explains novel variance in these harmful behaviors not captured by well-established personality and environmental risk factors for aggression.

## Introduction

Aggression is a serious public health concern and is associated with a variety of negative health outcomes, including substance misuse, suicide and chronic pain (Chida and Steptoe, 2009; McCloskey *et al.*, 2010; Hellmuth *et al.*, 2012). Aggressive behaviors are complex and multidimensional, encompassing a wide range of actions intended to inflict harm onto others (DeWall *et al.*, 2011). Decades of research on this topic have explored the etiology of aggression. However, few studies have examined the degree to which different levels of analysis, such as neurobiological, psychological and environmental factors, relate to aggressive behaviors in the same study. Furthermore, no previous published research has examined the unique contributions of these factors for explaining aggressive behaviors in multilevel models—information that would advance the understanding of the multidetermined nature of aggression. To address this gap, the present study situated aggression within a biopsychosocial framework by examining three levels of analysis—neurobiological (cortical thickness), psychological (pathological personality traits) and environmental (assaultive trauma exposure) contributors to aggression—in a high-risk community sample of adults.

Identifying the neuroanatomical underpinnings of aggression is potentially useful for elucidating mechanisms that contribute to the maintenance and proliferation of these harmful behaviors. Research implicates diminished cortical thickness and gray matter volumes in the prefrontal cortex (PFC) in the etiology of aggression (Raine *et al.*, 2000; Blair, 2016; Chester *et al.*, 2017; Ling *et al.*, 2019; Raine, 2019). This is consistent with the PFC’s role in emotion and behavior regulation. Structural and functional abnormalities in temporal cortices have also been associated with psychopathic traits, a constellation of personality features that demonstrate robust positive associations with aggressive behaviors in the literature (Blair, 2010; Garofalo *et al.*, 2020). For instance, Ly *et al.* (2012) found less thickness in the anterior temporal cortices of incarcerated adults with psychopathic traits than without psychopathic traits, providing indirect evidence of an association between aggression and cortical thickness. Given that neuroscience research has predominantly focused on understanding aggression in youth (Card and Little, 2006) or aggressive traits in adulthood (Yang and Raine, 2009; Lamsma *et al.*, 2017; Ling *et al.*, 2019; Raine, 2019), it is important to examine the generalizability of these findings for explaining adult aggression along a continuum of severity, a current gap in the literature (Wahlund and Kristiansson, 2009; Chester *et al.*, 2017; Lamsma *et al.*, 2017).

A second limitation of previous work is the tendency to study neurobiological correlates of aggression in isolation rather than integrating them into etiological models with other well-established risk factors. As highlighted in a recent meta-analysis by Lamsma *et al.* (2017), the neurobiological correlates of aggressive behavior have been inconsistent, which is likely in part caused by its multidetermined nature. Contextualizing neuroanatomical findings in broader biopsychosocial models can further the understanding of whether neuroanatomical markers, like cortical thickness, account for unique variance in aggression above and beyond the established risk processes or if neural markers simply reflect biological instantiations of known contributors (e.g. trauma and impulsivity). Similarly, realization of the translational significance of neural markers, such as their application in the neuroprediction of aggression, would rest on the ability of such metrics to improve upon the reliability and predictive validity of less onerous and expensive non-neuroimaging assessments (Poldrack *et al.*, 2018; Romero-Martínez *et al.*, 2019), which has yet to be established. Thus, etiological models will benefit from the inclusion of neurobiological markers of aggression if these metrics demonstrate incremental validity in relation to widely validated non-neuroimaging assessments of risk factors (Poldrack *et al.*, 2018).

Relative to neurobiological investigations, a great deal of attention has been devoted to understanding the contribution of personality traits to aggression. According to the General Aggression Model, repeated engagement with aggressive stimuli, including violent video games and media, along with positively reinforced aggression, is likely to foster the development of aggressive personality subtypes (Anderson and Bushman, 2002). Consistent with this model, meta-analyses find that personality traits, specifically low conscientiousness and low agreeableness, are strongly associated with aggression (Miller and Lynam, 2001, 2003; Jones *et al.*, 2011). Notably, personality traits and aggression evidence such associations consistently across both developmental stages and contexts (i.e. community samples, outpatient psychiatric patients, juvenile delinquents and prisoners) (Miller and Lynam, 2001; Bettencourt *et al.*, 2006; Jones *et al.*, 2011), underscoring the robustness of these relationships. Thus, low scores on conscientiousness and agreeableness may foster greater accessibility to aggressive emotional states, conferring risk for aggression. While these studies certainly advance our understanding of the role of personality in models of aggression, the relatively small effect sizes of these findings underscore the need to simultaneously consider how multiple factors contribute to the onset and maintenance of aggression.

Another recurrent finding in the literature is the ‘cycle of violence’ or the link between exposure to trauma in childhood and aggression perpetration in adulthood (Maxfield and Widom, 1996; Wilson *et al.*, 2009; Rasche *et al.*, 2016). Research implicates assaultive trauma types, such as physical abuse (Dutton and Hart, 1992), and repeated exposure to different forms of abuse as especially strong predictors of aggression in adulthood (Maschi *et al.*, 2008). Of note, recent findings implicate reductions in cortical thickness as a potential mechanism mediating the relationship between early violence exposure and later violence perpetration (Bounoua *et al.*, 2020). This work points to the importance of examining trauma type and, in particular, the co-occurrence of different trauma types, within neurobiological models of aggressive behavior.

Although research has identified neurobiological, personality and environmental processes that confer risk for aggression, no previous published research has examined the unique contributions of these factors in multilevel models—information that would advance understanding of the multidetermined nature of aggression. We sought to address this limitation by first examining whether neurobiological (cortical thickness), psychological (personality) and environmental (trauma) factors each explained variance in lifetime history of aggressive behaviors in an adult community sample. Given prior work showing that cortical thickness, personality and trauma exposure are interrelated (Sadeh *et al.*, 2015a,b; Bounoua *et al.*, 2020), we also tested the unique influence of the neurobiological, psychological and environmental factors in an integrative model of aggression. Results from these analyses will clarify whether these levels of analysis are redundant or explain non-overlapping variance in lifetime aggression. The significance of this approach is that it elucidates the relative importance of different types of risk processes for explaining aggressive behavior, which has yet to be established in the literature. Very little work has examined whether neurobiological metrics explain additional variance in aggressive behavior above and beyond other well-established psychological and environmental risk factors. Evaluating the relative importance of novel correlates of aggression, like cortical thickness, in models that incorporate psychological and environmental risk factors is important based on previous research demonstrating these levels of analysis are mutually influential (e.g. impulsivity personality traits and trauma exposure explain variation in cortical thickness).

Based on previous research, we hypothesized that aggression would inversely relate to cortical thickness in frontal and temporal cortices, as these regions have been implicated in a range of processes relevant to aggression (e.g. decision-making, emotion regulation and impulse control; Wahlund and Kristiansson, 2009; Blair, 2016; Ling *et al.*, 2019; Raine, 2019). Next, we hypothesized that the frequency of aggression would be positively associated with trait antagonism and disinhibition (Jones *et al.*, 2011) and exposure to assaultive trauma types (Dutton and Hart, 1992; Maschi *et al.*, 2008). Finally, given the limited data on integrative models of aggression, we examined the unique contributions of cortical thickness, personality traits and trauma exposure for explaining aggressive behavior simultaneously in a multilevel model.

## Methods

### Participants

The sample included 129 men and women aged 18–50 (*M*/s.d.* *= 30.7/8.3, 52.7% women) who completed a battery of self-report questionnaires and a neuroimaging protocol. The sample was diverse with respect to race, socioeconomic status and educational history. Participants identified primarily as White (50.0%) and Black/African American (37.5%), followed by Asian (7.8%) and Biracial (2.3%) (all other races were endorsed by <1%). A minority of participants identified as Hispanic/Latino (16.3%). The average household income for the sample was $49 248 per year (s.d.* *= $46 988), and the majority of participants came from communities with high rates of violent and non-violent crime based on available statistics (http://www.neighborhoodscout.com/de/Wilmington/crime on 1/20/2021). The highest grade level attained by the majority of participants was a high school diploma/General Educational Development (GED) (45.7%), followed by bachelor’s degree (17.8%), master’s degree (15.5%), associate’s degree (13.2%), less than grade 12 (7.0%), and doctorate or professional degree (0.8%).

Participants aged 18–50 who were fluent in English were recruited through the use of community flyers and online advertisements. Participants with current psychosis, a serious medical or neurological condition, history of head injuries with lasting effects or any magnetic resonance imaging (MRI) contraindications were excluded. An additional three participants were excluded based on incidental MRI findings and/or excessive motion.

### Procedures

The University of Delaware Institutional Review Board approved the current study (Protocol nos.: 1073423-17, 1361164-1). Written and oral consent was obtained from all individuals prior to participation in the study. The authors assert that all procedures contributing to this work comply with the ethical standards of the relevant national and institutional committees on human experimentation and with the Helsinki Declaration of 1975, as revised in 2008.

### Measures

#### Aggressive behaviors.

The self-report Risky, Impulsive, and Self-destructive behavior Questionnaire (RISQ; Sadeh and Baskin-Sommers, 2017) was used to assess the frequency of physically aggressive behaviors across the lifespan. Participants were asked to select the option that best describes the number of times they engaged in five examples of physically aggressive behaviors (e.g. ‘Gotten in a physical fight’ or ‘Attacked someone with a weapon, such as a knife or gun’) in their lives using this scale: 0 = 0, 1 = 1–10, 2 = 11–50, 3 = 51–100 and 4 = >100 acts. A total lifetime aggression score was created by summing responses on the five items (Cronbach’s alpha = 0.81), which showed good internal consistency. The rationale for using the binned options was to reduce the skewness of the items; however, the total lifetime aggression score was still positively skewed (2.00) and kurtotic (6.49). We applied Blom’s transformation to reduce the impact of outliers at the high end of the distribution as this transformation is uniquely suited for dealing with asymmetric distributions (Ayán and Díaz, 2008) and has been employed in previous work on aggression (Niv *et al.*, 2013; Pascual-Sagastizabal *et al.*, 2019). The RISQ has demonstrated reliable scales and convergent validity with other self-report measures of risky behavior, including aggressive behavior (Miglin *et al.*, 2019; Bounoua *et al.*, 2020; Estrada *et al.*, 2020).

#### Cortical thickness.

Data were collected at the University of Delaware using a Siemens 3T Magnetom Prisma scanner with a 64-channel head coil. A T1-weighted multiecho Magnetization-Prepared Rapid Acquisition with Gradient Echo (MPRAGE) anatomical scan (resolution = 1 mm^3^, Repetition Time (TR) = 2530 ms, Echo Time (TE) = 1.69, 3.55, 5.41, 7.27 ms) was collected to distinguish gray from white matter. The multiecho MPRAGE has the advantage of less distortion and higher contrast than standard MPRAGE sequences, resulting in more reliable cortical models (van der Kouwe *et al.*, 2008). A T2-weighted variable flip-angle turbo spin echo scan (resolution = 1 mm^3^, TR = 3200 ms, TE = 564 ms) was collected, which was used in FreeSurfer to better differentiate the gray matter–dura boundary. Segmentation of the cortical mantle and thickness of the cortical mantle at each vertex was calculated using the FreeSurfer v6 standard morphometric pipeline (Fischl *et al.*, 2002, 2004). Surface-based measurements of cortical thickness for all subjects were smoothed using Gaussian kernels of 10 mm full width at half maximum following previous similar analyses (Bernal-Rusiel *et al.*, 2010; Hyatt *et al.*, 2012).

#### Personality traits.

The Personality Inventory for Diagnostic and Statistical Manual of Mental Disorders Fifth Edition (DSM-5)-5-Brief Form-Adult is a 25-item self-report measure used to assess five pathological personality trait domains (American Psychiatric Association, 2013). Participants rated how much each item described them from 0 (Very False or Often False) to 3 (Very True or Often True). We focused on disordered personality traits that have been associated with aggressive behavior (Miller and Lynam, 2001; Jones *et al.*, 2011): Antagonism was assessed by summing five items (e.g. ‘I use people to get what I want.’; Cronbach’s alpha = 0.63) and Disinhibition was determined by summing five items (e.g. ‘I feel like I act totally on impulse.’; Cronbach’s alpha = 0.83). Each trait domain has a possible range of 0–15 points.

#### Trauma exposure.

The Trauma Screen from the Structured Clinical Interview for DSM-5 Revised Version stressor-related disorders module (First *et al.*, 2015) was used to assess lifetime exposure to 13 traumatic events. We calculated a total score that reflected the total number of types of assaultive trauma endorsed (Cronbach’s alpha = 0.71). These traumatic experiences included, but were not limited to physical abuse, assault, domestic violence and sexual violence.

### Data analysis

First, we examined the association of cortical thickness, personality traits and assaultive trauma separately with lifetime aggression using linear regression analyses. Biological sex, age, body mass index (BMI) and education level were entered as covariates of no interest in all analyses, based on previous associations with cortical thickness and/or aggression (Veit *et al.*, 2014; Medic *et al.*, 2016; Miglin *et al.*, 2019).

Pearson correlations were used to examine bivariate relationships. To assess associations between aggressive behavior and cortical thickness, we conducted vertex-wise analyses of the whole cortex. General linear models with frequency of lifetime aggression as the explanatory variable were conducted separately for each hemisphere using FreeSurfer’s QDEC software. The vertex-wise threshold was set at *P *< 0.01. To correct for multiple comparisons, we utilized a Monte Carlo simulation with 10 000 iterations and a cluster-based threshold of *P *< 0.05, correcting for the number of comparisons across both hemispheres. Hierarchical linear regressions were conducted in SPSS v26 with lifetime aggression regressed on covariates (block 1) and explanatory variables (block 2). A similar set of hierarchical linear regressions were conducted to test our integrative model with lifetime aggression regressed on our explanatory variables. We ruled out multicollinearity problems in the regression analyses, as evidenced by tolerance levels—all above 0.20 (Gaur and Gaur, 2006). All tests were two-tailed.

## Results

### Descriptive statistics

Participants reported a range of aggressive behavior across the lifespan, with 82.9% reporting at least some form of aggression and the average participant reporting 11–50 acts of lifetime aggression (binned *M*/s.d. = 2.7/2.5). In terms of disordered personality traits, average antagonism and disinhibition scores fell between the norms reported for clinical and non-clinical samples (Bach *et al.*, 2016). Overall, the sample reported a range of different types of traumatic events, averaging 2.1 types (s.d. = 1.81), with experiences of domestic violence (41.9%) and physical abuse (38.8%) endorsed most commonly.

Bivariate correlations are reported in Table 1. Participants who reported more aggressive acts were also older and had a higher BMI, on average, than those who reported less aggression. As expected, lifetime aggression correlated positively with cumulative exposure to different types of traumatic events. Lifetime aggression also demonstrated a positive correlation with antagonism and disinhibition.

**Table 1.** Bivariate associations between study variables

	*M*/s.d. or *N*/%	1	2	3	4	5	6	7
^1^Aggression frequency	2.61/0.91							
^2^Total assaultive trauma types	2.10/1.81	0.50^**^						
^3^Trait antagonism	2.84/2.66	0.33^**^	0.16					
^4^Trait disinhibition	4.41/3.21	0.20^*^	0.25^**^	0.55^**^				
^5^Age	30.68/8.25	0.34^**^	0.40^**^	−0.03	0.01			
^6^Female sex	68.00/52.71%	0.15	−0.07	0.11	−0.01	0.07		
^7^Education^a^	2.91/1.27	−0.19^*^	−0.17	−0.02	−0.11	0.10	−0.01	
^8^BMI	26.92/5.20	0.18^*^	0.17	0.02	−0.07	0.07	−0.03	−0.08

*Notes*: *N *= 129.

** *P* **< 0.01.

* *P* **< 0.05.

a Education *M/*s.d. scores were derived from the following scores: 1 = < 12th grade; 2 = high school diploma/GED; 3 = associate’s degree; 4 = bachelor’s degree; 5 = master’s degree; 6 = doctorate/professional. Average trait antagonism and disinhibition scores were compared to norms reported in clinical samples and non-clinical samples, with scores falling between the two population types (Bach *et al.*, 2016).

### Cortical thickness

**Fig. 1.**
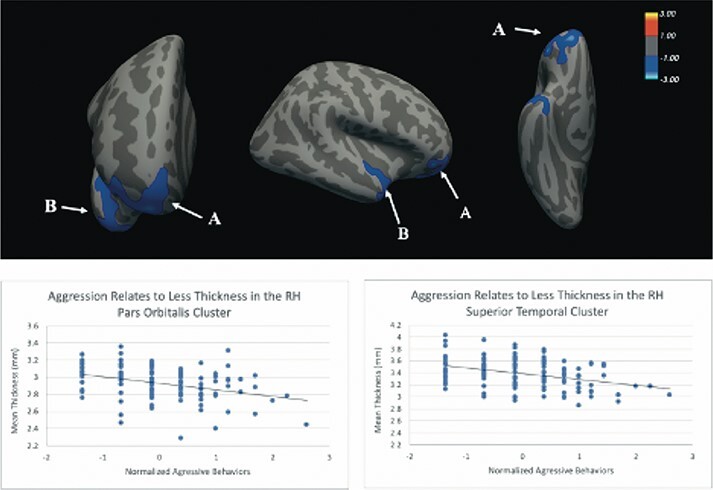
Aggression frequency relates inversely to cortical thickness in the right hemisphere. ROI A includes the IFG, pars orbitalis, lateral and medial OFC, frontal pole and rMFG. ROI B includes the superior temporal gyrus and temporal pole. Analyses were adjusted for age, sex, education level and BMI. All clusters survived Monte Carlo simulation correction for multiple comparisons across both hemispheres.

**Fig. 2.**
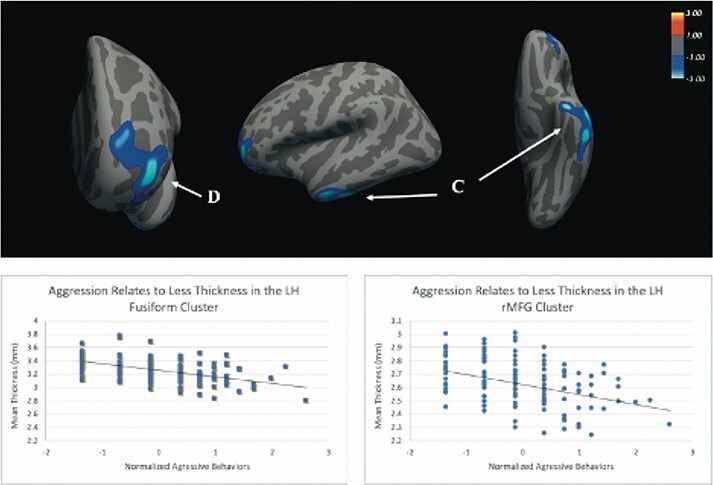
Aggression frequency relates inversely to cortical thickness in the left hemisphere. ROI C includes the fusiform, inferior temporal gyrus and middle temporal gyrus. ROI D includes the rMFG, superior frontal gyrus, IFG, pars orbitalis and lateral OFC. Analyses were adjusted for age, sex, education level and BMI. All clusters survived Monte Carlo simulation correction for multiple comparisons across both hemispheres.

In four clusters, cortical thickness decreased as the frequency of lifetime aggression increased (Table 2 and Figures 1 and 2). The first cluster peaked in right inferior frontal gyrus (IFG) pars orbitalis and spanned lateral/medial orbitofrontal cortex (OFC), frontal pole and rostral middle frontal gyrus (rMFG). The second cluster centered on right superior temporal gyrus and also included temporal pole. The third cluster peaked in left fusiform gyrus region and spanned inferior and middle temporal gyri. The fourth cluster peaked in left rMFG and included portions of superior frontal gyrus, IFG pars orbitalis and lateral OFC.

**Table 2.** Clusters showing a significant relationship between frequency of lifetime aggression and cortical thickness

Cluster No.	Hemisphere	Annotation	Peak *F*-value	Peak MNI (*x*, *y*, *z*)	No. of vertices	Cluster size (mm^2^)
A	R	IFG pars orbitalis lateral/medial OFC Frontal pole rMFG	−2.91	42.7, 40.0, −13.1	2085	1454.96
B	R	STG Temporal pole	−2.17	48.0, −2.3, 13.5	2026	1079.56
C	L	Fusiform gyrus ITG MTG	−3.89	−31.3, 1.4, −33.8	2088	1319.20
D	L	rMFG SFG IFG pars orbitalis Lateral OFC	−4.66	−32.1, 50.8, −3.5	1740	1251.50

*Note*: R = Right, L = Left , SFG = Superior frontal gyrus, ITG = Inferior temporal gyrus. Cluster A: Total *R*^2^ = 0.09**. Cluster B: Total *R*^2^ = 0.12**, Cluster C: Total *R*^2^ = 0.07**. Cluster D: Total *R*^2^ = 0.08**. **P < 0.01. *P < 0.05.

### Psychological and environmental phenotypes

Frequency of aggressive behaviors was positively associated with disordered personality traits (see Table 3), specifically antagonism and disinhibition. Frequency of aggressive behaviors was also positively associated with exposure to different types of assaultive trauma.

**Table 3.** Assaultive trauma and disordered personality traits in adulthood regressed on adult aggression severity

	Total assaultive trauma types	Trait antagonism	Trait disinhibition
Block 1			
Age	0.29^**^	−0.16	−0.05
Sex	−0.15	0.07	−0.04
Education	−0.13	0.06	−0.08
BMI	0.07	−0.04	−0.11
Block 2			
Aggression frequency	0.39^**^	0.39^**^	0.23^*^

*Note*: Values are standardized β values. Total assaultive trauma types: Step 1 *R*^2^ = 0.23**. ∆*R*^2^ = 0.12**. Total *R*^2^ = 0.35**. Trait Antagonism: Step 1 *R*^2^ = 0.01. Step 2 ∆*R*^2^ = 0.13**. Total *R*^2^ = 0.14**. Trait Disinhibition Step 1 *R*^2^ = 0.02. Step 2 ∆ *R*^2^ = 0.04*. Total *R*^2^ = 0.06*.

** *P* < 0.01.

* *P* < 0.05.

### Examination of an integrative model

We regressed lifetime aggression on all of the explanatory variables from the previous analyses that showed significant associations with aggression perpetration. This approach highlights the unique contributions of cortical thickness, personality and trauma exposure in explaining aggression. To reduce sample bias in our examination of cortical thickness, we first created an Region of Interest (ROI) mask for each cluster and extracted total thickness from all cortical parcellations included in the cluster using the Destrieux Atlas. Then, we entered these ROIs in separate linear regression analyses (to reduce multicollinearity among the thickness clusters) with the personality and trauma variables to examine the unique contributions of each type of variable for explaining aggression in an integrative model.

Results of these analyses revealed antagonism (β values > 0.28, *P* values < 0.01) and exposure to multiple types of assaultive trauma (β values > 0.41, *P* values < 0.001) were significant predictors of lifetime aggression in all models, whereas disinhibition was not (β values < −0.06, *P* values* *> 0.34). In addition, the cortical thickness clusters were also significant predictors of lifetime aggression. Specifically, the right IFG pars orbitalis (β = −0.17, *P* = 0.03),^1^ the right superior temporal gyrus and temporal pole (β* *= −0.20, *P* = 0.01),^2^ left fusiform gyrus (β = −0.15, *P* = 0.05)^3^ and left rMFG (β = −0.18, *P *= 0.02)^4^ all accounted for additional variance in aggression above and beyond trait antagonism and trauma exposure. Overall, the right IFG model [*R^2^*= 0.35, *F*(4, 122) = 16.18, *P* < 0.001], the right temporal model [*R^2^* = 0.36, *F*(4, 122) = 16.84, *P *< 0.001], the left fusiform model [*R^2^*= 0.34, *F*(4, 122) = 15.80, *P* < 0.001] and the left rMFG model [*R^2^* = 0.35, *F*(4, 122) = 16.50, *P* < 0.001] accounted for significant variance in lifetime aggression.

Based on evidence that the expression and correlates of aggression can vary by sex (Clauss *et al.*, 2017), we conducted supplemental analyses to examine whether the observed findings differed in men and women. Specifically, we reconducted the integrated regression analyses with sex as a moderator of the effects of trauma exposure, pathological personality traits and cortical thickness on lifetime frequency of aggression. These analyses produced no new significant findings.

## Discussion

The present study sought to contextualize adult aggression within a multilevel framework of biopsychosocial risk factors. To this end, we examined how neurobiological, psychological and environmental factors each accounted for unique variance in lifetime history of aggressive behaviors. At a neural level, findings showed that increased frequency of aggression was associated with less cortical thickness in regions spanning the prefrontal and temporal cortices. In addition, frequency of aggression was positively associated with antagonism and disinhibition, as well as experiences of multiple types of assaultive trauma across the lifespan. Finally, tests of integrative models revealed that all three levels of analysis (antagonism, experiences of assaultive trauma and cortical thickness in the bilateral clusters spanning the frontal and temporal regions) explained unique variance in lifetime aggressive behaviors. Together, these findings situate aggression within an integrative framework, providing a more holistic multilevel assessment of the biopsychosocial risk factors at stake in the maintenance of these behaviors.

### Aggression and cortical thickness

Vertex-wise analysis of the cortical mantle revealed that cortical thickness was related to frequency of aggressive behaviors in four clusters that survived correction for multiple comparisons (Figures 1 and 2). First, we found that frequency of aggressive behaviors was inversely associated with cortical thickness in the right and left prefrontal cortices. Bilaterally, more aggression was associated with less thickness in clusters that spanned pars orbitalis, rMFG, lateral OFC and adjacent frontal regions. Previous research has linked activation in the pars orbitalis region to inhibition of habitual responses to positive reinforcers (Matsubara *et al.*, 2004), suggesting that deficits in this area could contribute to difficulty overriding the reflexive reactions implicated in aggression. The OFC is also known to be important for reinforcement learning, and impaired OFC function is thought to instantiate the states of hyperarousal and dyscontrol seen in impulsive and reactive aggressors (Blair, 2004, 2016; Miller *et al.*, 2008). In addition, the rMFG, part of the dorsolateral PFC, is involved in executive control processes, like working memory, that are key for successful emotion and behavioral regulation in response to changing environmental contingencies. Taken together, the reductions in prefrontal cortical thickness found in our study converges with the broader literature on behavioral dysregulation. These results suggest an impaired capacity of executive control processes to regulate emotional states in arousing situations perpetuates aggressive outbursts.

In addition to reduced prefrontal thickness, we found greater aggressive behavior was associated with thinner cortex bilaterally in the temporal lobe. One cluster in the right hemisphere, spanning superior temporal gyrus and temporal pole, showed reductions in thickness as history of aggression increased. The right superior temporal gyrus has been implicated in social cognition and perception, perspective-taking, attention to emotion, and more recently, in forgiveness and empathy (Matsumoto *et al.*, 2001; Leslie *et al.*, 2004; Völlm *et al.*, 2006; Müller *et al.*, 2008). Thus, less thickness in these areas may increase aggression via deficits in perspective-taking and emotion perception. This interpretation would be consistent with prior research reporting thinning of temporal pole and superior temporal gyrus in psychopathic offenders (Ly *et al.*, 2012) and adults elevated on psychopathic traits (Yang and Raine, 2009). The left temporal cluster was located in the fusiform region and has been linked to the recognition of facial expressions (Yang and Raine, 2009). Failure to recognize and emotionally respond to facial or other signals of distress might contribute to a subsequent failure to inhibit behavior that leads to distress in others such as aggressive behaviors (Deeley *et al.*, 2006). In conjunction, the cortical thickness findings converge with prior work on aggression in clinical and forensic samples, indicating that a broad network of brain regions is associated with the manifestation of lifetime aggressive behavior.

### Relations with psychological and environmental phenotypes

Consistent with prior research (Miller and Lynam, 2003; Bettencourt *et al.*, 2006; Jones *et al.*, 2011; Anderson *et al.*, 2014; Hyatt *et al.*, 2019), we also found that aggression increased as levels of trait antagonism and disinhibition increased. In line with previous models of aggression, such as the Generalized Aggression Model, individuals with high levels of trait antagonism and/or disinhibition might have an elevated risk of attending to antisocial or hostile cues when confronted with different situations, thereby increasing the likelihood of aggressive interactions and ultimately reinforcing these behaviors (Allen and Anderson, 2017). Our findings further elucidate some of the mechanisms contributing to the persistence of aggressive behaviors. Namely, the underlying personality traits might uniquely predispose individuals to aggress onto others due to enhanced accessibility to aggressive attitudes and emotions. Further, preliminary findings suggest higher scores of conscientiousness, or the complement of disinhibition, are linked with greater volume in pars orbitalis (DeYoung *et al.*, 2010), a region where thickness was inversely associated with aggression in our sample. In addition, DeYoung *et al.* (2010) also found a positive association between agreeableness (the counter to antagonism) and volume in the fusiform region. Integration of these pathological personality traits into general models of aggression and identifying their neurobiological correlates may facilitate the identification of proximal risk factors such as substance use or psychological distress that were not examined in this study.

Although these personality traits are thought to remain relatively stable across the life span, evidence suggests that they also interact with environmental factors such as trauma. Consistent with prior research, experiencing multiple types of assaultive trauma in our sample was linked with greater engagement in aggressive behavior. Past work suggests that certain personality characteristics like antisocial traits, neuroticism and openness may predispose individuals to a higher incidence of stressful interpersonal life events into adulthood, possibly through individual differences in environment selection (Van Os *et al.*, 2001; Kandler *et al.*, 2012; Pos *et al.*, 2016). In this way, trait-level vulnerabilities might place individuals at a greater risk of experiencing multiple types of trauma and consequently exhibiting more aggressive behaviors. However, longitudinal studies are needed to parse the interactive effects of personality traits and environmental stress over time, including in relation to the perpetration of aggression.

### Biopsychosocial models of aggression

Establishing the relative contributions of different types of risk factors for explaining aggression is an important, yet understudied, research question. To examine the explanatory power of different types of risk processes, we tested general linear models of lifetime aggression that evaluated the unique contributions of the neurobiological, psychological and environmental variables that we linked with aggression. Trauma exposure, trait antagonism and thickness in each of the ROIs showed unique associations with aggression in the integrative models we conducted. These findings indicate that trauma exposure, pathological personality traits and cortical thickness provided non-overlapping information about perpetrating aggression, which is a novel finding that advances prior work that has investigated these risk processes separately. Although factors contributing to the persistence of aggression into adulthood have been examined in clinical and youth samples longitudinally, the current findings extend the literature by contributing to a general biopsychosocial model of aggression in a community sample.

## Strengths and limitations

 The findings of the present study must be interpreted within the context of its limitations. Firstly, the explanatory variables were assessed cross-sectionally, inherently limiting our ability to make causal inferences about their relationships or rule out other variables at stake. For example, we were unable to determine whether genetic vulnerabilities were related to the brain features and aggressive behaviors we identified in adulthood. This would be consistent with previous epigenetic work highlighting some shared genetic basis for aggression (Waltes *et al.*, 2016). Although this was outside the scope of our project, longitudinal data are needed to elucidate the temporal ordering of these variables, assessing the unique contributions of genetic and environmental risk (Toth and Cicchetti, 2013; Teicher and Samson, 2016; Bounoua *et al.*, 2020; Carlisi *et al.*, 2020). In addition, aggressive behaviors and assaultive trauma were assessed retrospectively, which could be more prone to recall bias. However, prior research examining retrospective reports of childhood adversity suggest that retrospective reports of serious and easily operationalized events can be considered valid in the research context (Hardt and Rutter, 2004). Research on differences in the etiology of aggression in women and men is a relatively understudied topic in this field (Burt, 2009; Burt *et al.*, 2018) and one that requires closer examination. Although we tested for differences in the observed correlates of lifetime aggression as a function of sex, we were likely underpowered to detect such effects. An interesting question to address for future research is whether including potentially sex-specific risk factors for aggression improves the explanatory power of multilevel models of the perpetration of aggressive behaviors. Finally, given the focus on physical aggression in this study, it is important to recognize that the results may likely not be generalizable to other forms of aggression (e.g. relational or verbal aggression). Although the perpetration of different forms of aggression often co-occurs (Allen and Anderson, 2017), the relevance of the present findings for explaining other types of aggression is a question that requires further study.

In terms of strengths, the study took a multidimensional approach by testing biological, psychological and environmental risk factors related to aggression perpetration. Although prior research has examined these dimensions individually, few studies have assessed these risk factors concurrently. Furthermore, the community sample we drew from was also uniquely suited to answer our research question. Namely, the sample was fairly large for a neurobiological study. In addition, whereas most of the literature investigating the neurobiological correlates of aggression have focused on forensic or clinical samples, the use of a community sample allowed us to extend the current findings to a more general neurobiological model of aggressive behaviors. Taken together, the results derived from our sample provide an extensive picture of aggression, less prone to error due to small sample size or a disproportionate emphasis on severe forms of aggression. Furthermore, while prior neuroimaging work has mainly focused on ROI analytic approaches, the present study employed a whole-cortex analysis, which further strengthens the robustness of our findings and allowed for the potential identification of neural regions not previously cited in the literature.

 In summary, the present study provides a unique biopsychosocial account of aggression by examining psychological, environmental and neurobiological correlates of these behaviors. Future research should further test this integrative model of aggression by examining the interrelations of the components. This type of study would permit a deeper understanding of the temporal relationship of the identified factors and could be used to isolate more proximal risk candidates for the onset and maintenance of aggressive behaviors.

## Footnotes

1 The right pars orbitalis ROI consisted of the following FreeSurfer labels: orbital sulci, orbital, gyri, frontomarginal gyrus (of Wernicke) and sulcus, straight gyrus, suborbital sulcus, superior frontal gyrus, and transverse frontopolar sulci.

2 The right superior temporal and temporal pole ROI consisted of the following FreeSurfer labels: temporal pole, planum polare of the superior temporal gyrus, inferior segment of the circular sulcus of the insula and lateral aspect of the superior temporal gyrus.

3 The left fusiform ROI consisted of the following FreeSurfer labels: middle temporal gyrus, inferior temporal gyrus, temporal pole and inferior temporal sulcus.

4 The left rMFG ROI consisted of the following FreeSurfer labels: frontomarginal gyrus (of Wernicke) and sulcus, orbital gyri, orbital, sulci, middle frontal gyrus, transverse frontopolar gyri and sulci, and superior frontal gyrus.
